# A Meta-Analysis Study to Infer Voltage-Gated K^+^ Channels Prognostic Value in Different Cancer Types

**DOI:** 10.3390/antiox12030573

**Published:** 2023-02-24

**Authors:** Beatrice Angi, Silvia Muccioli, Ildikò Szabò, Luigi Leanza

**Affiliations:** Department of Biology, University of Padua, 35121 Padua, Italy

**Keywords:** Shaker type Kv channels, cancer, melanoma, lung cancer, survival, Kv1.3, Kv1.5, Kv1.2

## Abstract

Potassium channels are often highly expressed in cancer cells with respect to healthy ones, as they provide proliferative advantages through modulating membrane potential, calcium homeostasis, and various signaling pathways. Among potassium channels, Shaker type voltage-gated Kv channels are emerging as promising pharmacological targets in oncology. Here, we queried publicly available cancer patient databases to highlight if a correlation exists between Kv channel expression and survival rate in five different cancer types. By multiple gene comparison analysis, we found a predominant expression of KCNA2, KCNA3, and KCNA5 with respect to the other KCNA genes in skin cutaneous melanoma (SKCM), uterine corpus endometrial carcinoma (UCEC), stomach adenocarcinoma (STAD), lung adenocarcinoma (LUAD), and lung squamous cell carcinoma (LUSC). This analysis highlighted a prognostic role of KCNA3 and KCNA5 in SKCM, LUAD, LUSC, and STAD, respectively. Interestingly, KCNA3 was associated with a positive prognosis in SKCM and LUAD but not in LUSC. Results obtained by the analysis of KCNA3-related differentially expressed genes (DEGs); tumor immune cell infiltration highlighted differences that may account for such differential prognosis. A meta-analysis study was conducted to investigate the role of KCNA channels in cancer using cancer patients’ datasets. Our study underlines a promising correlation between Kv channel expression in tumor cells, in infiltrating immune cells, and survival rate.

## 1. Introduction

Potassium channels that allow the selective flux of K^+^ ions through biological membranes down their electrochemical gradient comprise more than 70 proteins that display diverse regulation, functions, and tissue distribution. Among them, the eight Kv1 *Shaker* subfamily members (Kv1.1-Kv1.8 encoded by KCNA1, KCNA2, KCNA3, etc (KCNA10 for Kv1.8)) of the voltage-gated potassium channels (Kv channels) https://www.guidetopharmacology.org/GRAC/FamilyIntroductionForward?familyId=81 (accessed on 18 January 2023) [1] have been extensively studied during the past decades for the important roles they exert in the regulation of cell cycle progression, apoptosis, and migration in both healthy and pathological cells. Several studies found a correlation between the activity and expression of various potassium channels and the progression of different kinds of cancers [recently reviewed in [2]. However, to our knowledge, no systematic study using publicly available databases obtained from patient samples examined whether the expression of the *Shaker*-type potassium channels was altered and whether it correlated with prolonged or decreased survival rate. Given the availability of specific pharmacological modulators of the *Shaker* potassium channels [1] and considerable promising in vitro and in vivo preclinical data, such information might be useful in understanding whether pharmacological targeting of these ion channels may be of benefit.

Previous data pointed out that Kv1.1 was expressed in the breast cancer cell line MCF-7, and its blockade by Dendrotoxin (DTX) (10 nM) could reduce proliferation by 30% [3]. A high expression of the channel was observed in cervical cancer patients, correlating with poor prognosis and Kv1.1 silencing, which decreased the proliferation of HeLa cells [4]. DTX suppressed lung adenocarcinoma growth in vivo [5] and reduced the proliferation of even chemo-resistant non-small cell lung cancer (NSCLC) cells both in vitro and in vivo [6]. The specific inhibition of Kv1.1 by KAaH2 toxins repressed the proliferation of glioblastoma [7]. Interestingly, aberrant hypermethylation in the KCNA1 promoter region was observed in more than 200 colorectal cancer patients who held biomarker potential [8].

As to Kv1.2, much less information is available since this channel has been studied mainly in the context of pain. Its expression was barely detectable in a panel of gastric cancer cell lines [9]. However, this O_2_-sensitive channel was found to play a critical role in the hypoxia-induced depolarization of pheochromocytoma PC12 cells [10], but no follow-up studies were reported.

An important role for the ROS-modulated Kv1.3 [11] was instead well documented, both in cancer cells (for reviews see, e.g., [12,13]) and in some of the immune cells constituting the tumour microenvironment (TME) and actively modulating tumour progression [14]. The injection of Margatoxin (MgTx), an inhibitor of Kv1.3 (but also of Kv1.1 and Kv1.2 [15]), directly into the xenograft of the A549 human lung adenocarcinoma cell line at 1 nM concentration, inhibited tumor growth in a nude mice model [16]. Blockade of Kv1.3 by the most specific toxin inhibitor; Shk blunted the activation-induced proliferation of malignant T cells in Sezary syndrome, which express high levels of functional Kv1.3 [17]. An enhanced expression of Kv1.3 has also been observed in resected human pancreatic ductal adenocarcinoma [18] and in primary B cells from chronic lymphocytic leukemia patients [19]. In both cases, the modulation of Kv1.3 significantly decreased the tumor burden. Notably, Shk has recently become available orally as an engineered probiotic [20]. The study underlined its efficacy against autoimmune disease, but it would be worthwhile to explore whether such an administration route of Shk may have beneficial effects in the context of cancer. On the other hand, the modulation of the channel by pharmacological means in the TME immune cells might also be useful. For example, the cajanine derivative LJ101019C was shown to increase Kv1.3 activity and expression, leading to the proliferation and activation of natural killer (NK) cells, which are so important for anti-tumour immunity [21].

Regarding Kv1.4, the KCNA4 methylation pattern increased along with tumor grade progression and the predicted poor survival of glioma patients [22]. KCNA4 methylation was also enhanced in gastric cancer patients [23]. Whether KCNA4 hypermethylation correlates with high expression of the channel and the effects of altered expression/functions in cancer cells still await clarification.

As to Kv1.5, this O_2_-sensitive channel [24] has been shown to be upregulated in many types of tumors, where it promotes proliferation and metastatic tissues. High expressions of Kv1.5 correlated with leiomyosarcoma proliferation and aggressiveness [25] and were found in gastric and colorectal carcinoma specimens [26,27]. However, the expression of Kv1.5 shows an inversed correlation with malignancy in some gliomas and non-Hodgkin’s lymphomas [13,28]. Silencing Kv1.5 expression significantly inhibited proliferation and induced cell cycle arrest at the G0/G1 phase in osteosarcoma [29] but supported Ewing sarcoma cell proliferation [30]. Similarly to Kv1.3, Kv1.5 is also expressed in macrophages and is likely also in the tumor-associated macrophage population [31].

Kv1.6 was found to be expressed in prostate cancer cells [32], although to a lesser extent than Kv1.3. To our knowledge, studies regarding Kv1.7 and Kv1.8 expressions in cancer have not yet been performed.

Altogether, a conclusive general picture regarding the involvement of Shaker-type channels in promoting cell proliferation in cancer cells is still lacking. In addition, it has to be underlined that Kv1.1, Kv1.3, and Kv1.5 are present not only in the plasma membrane but also in mitochondria in several types of cells [33,34,35,36]. When the channel is found in the plasma membrane, usually it is expressed also in the mitochondria [33]. While the plasma-membrane channels modulate proliferation, the mitochondrial ones are linked to apoptosis regulation [37]. We also have to point out that Kv1.3 (and possibly other Shaker channels as well) can apparently also promote proliferation independently of its ion-conducting properties (see, e.g., [38,39]).

In the present study, we explored the publicly available TCGA database in order to systematically investigate the correlation between Shaker-type potassium channels in some types of cancers (with the highest Shaker channel mutational load) and patients’ survival.

## 2. Materials and Methods

### 2.1. cBioPortal

The cBioPortal for Cancer Genomics (http://www.cbioportal.org, accessed on 18 January 2023) is a repository of cancer genomics datasets [40,41]. We investigated genetic alteration frequencies of *KCNA* genes using the TCGA Pan-Cancer Atlas dataset, collecting data from 32 human cancers (10967 samples in total).

### 2.2. Gene Expression Profiling Interactive Analysis (GEPIA)

GEPIA (gene expression profiling interactive analysis) (http://gepia.cancerpku.cn/, accessed on 18 January 2023) is an interactive web application for gene expression analysis based on 9736 tumors and 8587 normal samples [42]. This platform was also used to analyse the expression of *KCNAs* in selected tumours and their effects on overall survival by means of the Kaplan–Meier analysis tool. Samples were divided between high and low *KCNAs* expression groups according to the quartile *KCNAs* mRNA levels to analyse survival rate (group cutoff selected quartile, cutoff-high (%): 75; cutoff-low (%): 25).

### 2.3. Survival Genie

To assess tumour infiltration according to *KCNAs* expression, Survival Genie software was employed (https://bbisr.shinyapps.winship.emory.edu/SurvivalGenie/, accessed on 18 January 2023). Survival Genie is an open source that contains 53 datasets of 27 distinct malignancies from 11 different cancer programs relating to adult and pediatric cancers [43].

### 2.4. UALCAN Database Analysis

To determine the clinical value of *KCNA3* and *KCNA5* expression levels, we used the UALCAN database (http://ualcan.path.uab.edu, accessed on 18 January 2023). UALCAN is an interactive and user-friendly web source that helps in analyzing cancer OMICS data and correlates them with clinicopathological features [43]. UALCAN was employed to evaluate the co-expression between KCNA3 and KCNA5 transcript levels with several clinical signatures or risk factors such as the absence or presence of tumor metastasis, nodal metastasis status, age, sex, weight, tobacco consumption, and *H. pylori* infections in cancer patients. The cancer stage was stratified between healthy tissues (“Normal”) and tumor stage (Stage 1 to stage 4). Patients’ weight was ranked based on BMI (body mass index): Normal weight (18.5 ≤ BMI ≤ 25), Extreme weight (25 ≤ BMI ≤ 30), Obese (30 ≤ BMI ≤ 40), and Extreme obese (BMI ≥ 40).

### 2.5. LinkedOmics

The LinkedOmics database (http://www.linkedomics.org/login.php, accessed on 18 January 2023) is a web tool that is useful for conducting cancer-associated multi-dimensional analyses deriving data from the TCGA datasets within and across 32 cancer types [44]. The LinkFinder module of LinkedOmics was used to study differentially expressed genes in correlation with KCNA3 and KCNA5 in the TCGA SKCM (*n* = 371), LUAD (*n* = 515), LUSC (*n* = 501), and STAD (*n* = 415) Firehorse cohorts perform pathway and network analyses. The results were statistically analyzed using Spearman’s correlation coefficient. GO enrichment analysis was used to perform GO analyses for the biological processes (BP). Graphs to analyze the correlation between gene expressions were also retrieved employing LinkedOmics.

### 2.6. Tumor Immune Estimation Resource (TIMER)

TIMER (tumor immune estimation resource) is an in silico web platform to analyze interactions between gene expression in tumors and infiltrating immune cell-types [45]. This tool collects data from 32 different tumor-types retrieved from the TCGA dataset, gathering gene expression profiles measured with RNA-seq or microarray to evaluate the abundance of the different immune infiltrates within the tumor microenvironment. KCNA3 mRNA levels and their association with infiltrated immune cells (B cells, CD4+T cells, CD8+T cells, Neutrophils, Macrophages, and Dendritic Cells) were assessed in overall SKCM, LUAD, and LUSC.

### 2.7. Statistical Analysis

Statistical analysis was performed using the CbioPortal, GEPIA, UALCAN, LinkedOmics, SurvivalGenie, and TIMER databases. Differences were examined for significance as per the figure legend specifications. Data were normalized as transcripts per kilobase million (TPM) values. Data were shown as the mean ± standard deviation. Statistical analyses were conducted using a t-test. Error bars represent SD. *, **, *** indicated *p*-value < 0.05, 0.01, 0.001, respectively. Kaplan–Meier analyses were plotted based on Fisher’s exact test (F-test). Values were normalized as TPM. Statistics of the survival analyses were performed using an F-test. The top 50 positively KCNA3 and KCNA5 correlating genes were mapped using the TCGA SKCM, LUAD, LUSC, and STAT dataset on the LinkedOmics database, according to their ranking and based on their Z-score through Spearman’s correlation analysis. The top 1000 KCNA3 and KCAN5 positively co-expressed genes were used for GO analysis obtained through Gene Ontology Enrichment Analysis performed on LinkedOmics, based on their *p*-values. Two Pearson correlation analyses between KCNA3 transcript levels and immune activation markers were retrieved from LinkedOmics using the SKCM, LUAD, and LUSC datasets. Data were plotted as scatter plots based on the Spearmans’ correlation coefficient (* *p* < 0.05; ** *p* < 0.01; *** *p* < 0.001). On the other hand, a correlation between the *KCNA3* level of expression and immune infiltration of cell types was obtained overall by SKCM, LUAD, and LUSC datasets.

## 3. Results

### 3.1. KCNAs Alterations and Expression in Human Cancers

**Figure 1:**
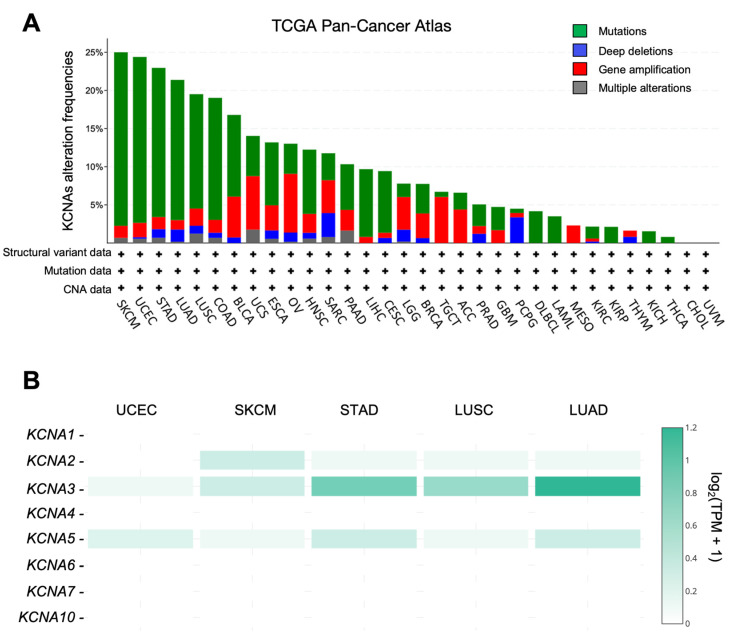
Genomic alteration frequencies and differential expression of KCNAs family in human cancers. (**A**) Mutation frequencies of KCNAs in 32 cancer studies were retrieved from cBioPortal (TCGA Pan-Cancer Atlas dataset). Cancer names list: ACC: Adrenocortical carcinoma; BLCA: Bladder Urothelial Carcinoma; BRCA: Breast invasive carcinoma; CESC: Cervical squamous cell carcinoma and endocervical adenocarcinoma; CHOL: Cholangio carcinoma; COAD: Colon adenocarcinoma; DLBC: Lymphoid Neoplasm Diffuse Large B-cell Lymphoma; ESCA: Esophageal carcinoma; GBM: Glioblastoma Multiforme; HNSC: Head and Neck squamous cell carcinoma; KICH: Kidney Chromophobe; KIRC: Kidney Renal Clear Cell Carcinoma; KIRP: Kidney renal papillary cell carcinoma; LAML: Acute Myeloid Leukemia; LIHC: Liver hepatocellular carcinoma; LGG: Brain Lower Grade Glioma; LUAD: Lung adenocarcinoma; LUSC: Lung Squamous Cell Carcinoma; MESO: Mesothelioma; OV: Ovarian serous cystadenocarcinoma; PAAD: Pancreatic adenocarcinoma; PCPG: Pheochromocytoma and Paraganglioma; PRAD: Prostate adenocarcinoma; SARC: Sarcoma; SKCM: Skin Cutaneous Melanoma; STAD: Stomach adenocarcinoma; TGCT: Testicular Germ Cell Tumors; THCA: Thyroid carcinoma; THYM: Thymoma; UCEC: Uterine Corpus Endometrial Carcinoma; UCS: Uterine Carcinosarcoma; UVM: Uveal Melanoma. (**B**) KCNAs multiple gene comparison was performed for UCEC, SKCM, STAD, LUSC, LUAD tumours using TCGA and GTEx datasets and using the GEPIA database. Data were normalized as transcripts per kilobase million (TPM) values. TPM values were converted to log2-normalized transcripts per million [log2(TPM + 1)].

The abnormal expression of potassium channels has been documented in many tumours and can be caused by the presence of mutations at the genomic level [46]. We evaluated the overall genetic alterations in all KCNA genes (KCNA1, KCNA2, KCNA3, KCNA4, KCNA5, KCNA6, KCNA7, KCNA10) using the TCGA Pan-Cancer Atlas dataset, collecting data from 32 human cancers (10,953 patients in total). Using the cBioportal Database, genomic alterations were classified into five categories per mutation (truncating mutations, in-frame mutations, or missense mutations), deep deletions (homozygous deletions for non-aneuploidy cases), gene amplification, structural variants, and multiple alterations [40]. The Cancer type summary representation revealed the distribution of KCNAs genomic alterations in the TCGA PanCancer cohorts (Figure 1A). Mutations in KCNA genes were present in 12% of queried patients. Results showed that the five cancer types with the highest alteration frequencies were skin cutaneous melanoma (SKCM), uterine corpus endometrial carcinoma (UCEC), stomach adenocarcinoma (STAD), lung adenocarcinoma (LUAD), and lung squamous cell carcinoma (LUSC), in which the overall mutation frequency was ranging from 25.01% for SKCM to 19.51% for LUSC. In particular, most of the genetic alterations in KCNA genes were determined through the presence of truncating mutations, in-frame mutations, or missense mutations, whereas amplifications, multiple alterations, or deep deletions were much less frequent. From the analysis of the single mutations on all *KCNA* genes, it was not possible to reveal any correlation between the presence of a certain mutation and the incidence of a specific cancer type. Likewise, these high-frequency mutations are not expected to change channel functions based on our current knowledge regarding the structure–function relationship of Kv channels.

Given this background, we explored the expression profile of KCNAs in five tumour types that showed higher KCNA mutation rates using the publicly available GEPIA database (gene expression profiling interactive analysis) [42]. The multiple gene comparison analysis showed that SKCM, UCEC, STAD, LUAD, and LUSC were overall characterized by a predominant expression of KCNA2, KCNA3, and KCNA5 with respect to the other KCNA genes (Figure 1B). Interestingly, KCNA3 is the most abundantly expressed KCNA gene in SKCM, STAD, LUSC, and LUAD.

With KCNA2, KCNA3, and KCNA5 being the most abundant transcripts of the *Shaker* gene family in SKCM, UCEC, STAD, LUSC, and LUAD, we compared their expression levels in both tumour and normal samples. The KCNA2 mRNA level does not significantly differ in controls compared to tumoral samples in any of the considered cancer types (Figure S1A). On the contrary, KCNA3 mRNA levels were significantly reduced in LUSC samples with respect to normal controls. No significant difference in KCNA3 expression can be observed instead in UCEC, STAD, LUAD, and SKCM (Figure S1B). As regards the KCNA5 transcript, UCEC, LUAD, and LUSC tumours displayed a statistically significant decrease in the expression of this gene with respect to normal controls, while no differences were present for SKCM and STAD (Figure S1C).

Overall, these data highlighted SKCM, UCEC, STAD, LUSC, and LUAD to be the five cancers with the highest KCNAs mutation frequency and revealed KCNA2, KCNA3, and KCNA5 to be the most expressed KCNA family genes in these tumours.

### 3.2. KCNA3 and KCNA5 Expression Impacts on Patients’ Survival

**Figure 2:**
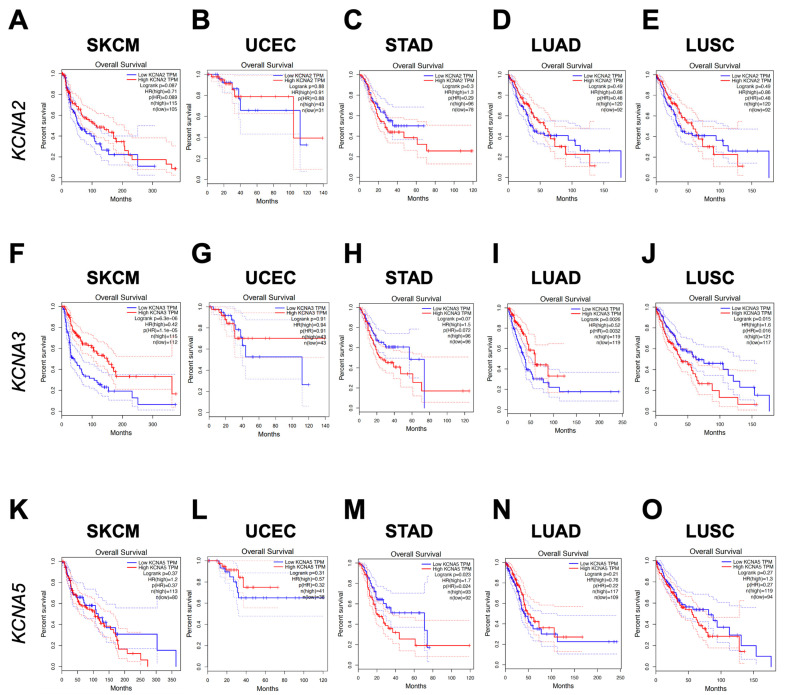
Kaplan–Meier curves based on KCNAs expression in patients. Survival plots based on KCNA2 (**A**–**E**), KCNA3 (**F**–**J**), and KCNA5 (**K**–**O**) expression level in represented tumours were obtained through Kaplan–Meier analysis by sorting samples for high and low KCNA expression groups according to the quartile on GEPIA. The percentage of survival is plotted, and *p*-values are shown as per figure specifications, respectively.

To explore the prognostic significance of *KCNA2, KCNA3,* and *KCNA5* expression in cancer patients, we took advantage of the Kaplan–Meier analysis by sorting samples for high and low KCNAs expression according to the quartile KCNAs mRNA level. This analysis revealed a prognostic value for KCNA3 in SKCM, LUAD, and LUSC (Figure 2B) and of KCNA5 in STAD (Figure 2C). Indeed, high *KCNA3* expression correlated with increased overall survival in SKCM patients (long-rank *p* = 6.3 × 10^−6^ and in LUAD patients (long-rank *p* = 0.0026) (Figure 2F,I). On the contrary, a high *KCNA3* mRNA level was associated with decreased survival in LUSC patients (long-rank *p* = 0.015) (Figure 2J). It is worth mentioning that LUSC was the only tumour analysed in which the *KCNA3* transcript significantly differed between the tumour and normal samples (Figure S1B). In STAD and UCEC patients, *KCNA3* expression showed no clear correlation with overall survival (Figure 2G,H). High *KCNA5* expression correlates with decreased survival rates in STAD patients (long-rank *p* = 0.023) (Figure 2M), while it has no association with survival in SKCM, UCEC, LUAD, and LUSC cancers (Figure 2K,L,N,O). Contrary to *KCNA3* and *KCNA5*, *KCNA2* has no prognostic value for any of the considered cancers (Figure 2A–E). Taken together, these data show the prognostic role of *KCNA3* and *KCNA5* in SKCM, LUAD, LUSC, and STAD, respectively.

However, the level of *KCNAs* transcripts in the different tumours and the difference in *KCNAs* expression between normal and tumour tissues do not provide an explanation for the effect that these genes play on survival. This indicates that other factors, rather than gene expression, need to be considered.

### 3.3. Clinical Features and Prognostic Value of KCNA3 and KCNA5 in Cancer

**Figure 3:**
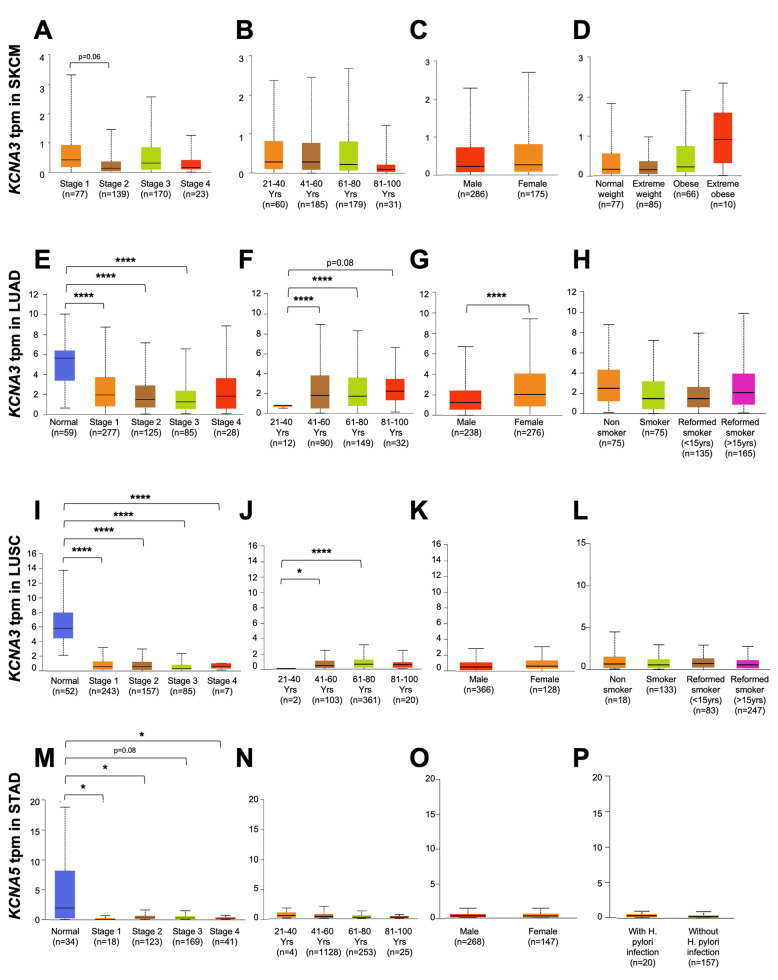
Clinical characterization and prognostic value of KCNAs in cancer. Boxplots represent the relationship between *KCNAs* mRNA expression with tumor staging (**A**,**E**,**I**,**M**), age (**B**,**F**,**J**,**N**), sex (**C**,**G**,**K**,**O**), and distinct risk factors [i.e., BMI (**D**), tobacco consumption (**H,L**), and h. pylori infection (**P**)] in patients affected by different cancer types, using the TCGA databases on UALCAN. Data were normalized as transcripts per kilobase million (tpm). The considered ion channels and cancer-types are reported on the Y-axis. Statistical significance was calculated using t-tests and are specified with asterisks (* *p* < 0.05, **** *p* < 0.0001). Data were shown as the mean ± standard deviation. Error bars represented SD.

To better understand the prognostic function of KCNAs channels that are significantly related to survival in the tumors considered in Figure 2 (SKCM, LUAD, and LUSC for KCNA3 and STAD for KCNA5, respectively), we decided to investigate the relationship between channel expression and the pathological-clinical features of malignancies. To this end, we determined whether the expression of KCNA3 and KCNA5 correlated with tumor stage progression, age, sex, and some specific cancer risk factors (Figure 3A–P).

Considering KCNA3, it is interesting to note that its expression tends to significantly decrease with tumor stage progression compared to normal tissues in both lung tumor types which it associates with patient prognosis (Figure 3E,I). In these cases, the *KCNA3* mRNA level is three-fold lower at LUAD stage 4 and over 16-fold lower at LUSC stage 4 compared to the channel’s expression in healthy tissues (“Normal”).

It is well known that the incidence of all types of cancer steadily increases with advancing age (National Cancer Institute). Indeed, aging is considered a generic risk factor for all types of cancers [47]. Noticeably, *KCNA3* expression displays an increase with advancing age both in LUAD and LUSC (Figure 3F,J). The same is not true for SKCM, where there is no clear correlation with age, probably since the channel’s expression levels in melanoma are significantly lower than those in lung cancer in terms of transcripts per million (tpm), so differences in expression may not be appreciable.

*KCNA3* expression also does not correlate to sex, except for LUAD, in which it is more expressed in females (Figure 3G). Numerous pieces of evidence underline the crucial role of obesity in tumor development and progression. Recently, multiple sources have demonstrated a close association between body weight gain and melanoma incidence [48]. Interestingly, the expression of KCNA3 shows a growing trend, although not significantly, correlating with the rise in body mass index (BMI) (Figure 3D).

Nevertheless, smoking represents the main risk factor for the onset of lung cancer [49,50]. Still, no relevant correlation between *KCNA3* expression and smoking habits has been reported (Figure 3H,L).

As for KCNA5, the expression of the ion channel in STAD patients drastically and significantly decreases in the different tumor stages compared to healthy tissues (Figure 3M). However, no correlations were found between *KCNA5* expression levels and other risk factors, including *H. pylory* infection (Figure 3P): one of the main risk factors for determining the onset of gastric tumors [51].

### 3.4. Expression of Known Interactors of Plasma-Membrane-Located Kv1.3 Do Not Correlate with KNCA3 Expression in Cancer

Next, since Kv1.3 is the channel mostly affecting patients’ survival in the considered malignancies, we investigated the correlation between KCNA3 expression and its known interactors. Indeed, Kv1.3 has been shown to interact with different proteins that are involved in signaling pathways that are linked to proliferation and migration. Therefore, we checked whether the expression level of KCNA3 correlated with that of its interactors to eventually associate the co-expression level with patients’ survival. The tested interactors are β1-integrin encoded by ITGB1 [52], cortactin (CTTN) (an SH3 domain-containing F-actin binding protein) [53], the PDZ family proteins DLG4 and DLG1 (also called PSD95 and SAP97, respectively) [54,55], the regulatory protein KCNE4 [56] as well as caveolin (CAV1) [57], and Sec24A from COPII [58]. As shown in Table 1, although a significant correlation between the expression of KCNA3 and of some of the interactors could be observed in all three types of cancers, the only protein whose expression negatively correlated with KCNA3 in SKCM and LUAD but not in LUSC, was cortactin. The knockdown of cortactin was shown to decrease the actin-based immobilization of Kv1.3 [53]. Interestingly, in melanoma patients, high levels of cortactin expression are correlated with poor disease-specific survival [59]; this is possibly because cortactin phosphorylation is a key step during invadopodia maturation and migration. Whether and how Kv1.3 expression regulates CTNN expression is unknown.

### 3.5. Identification and Analysis of the KCNA3 Differentially Expressed Genes (DEGs) in Cancer

**Figure 4:**
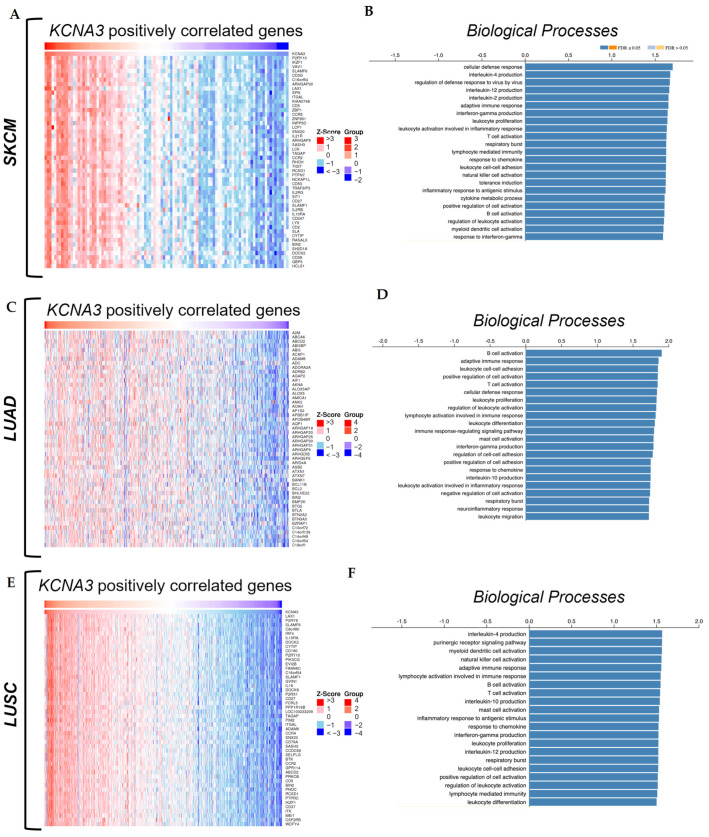
KCNA3 correlated differentially expressed genes and related pathways. The top 50 positively KCNA3 co-expressed genes were mapped using the TCGA SKCM (**A**,**B**), LUAD (**C**,**D**), and LUSC (**E**,**F**) datasets in the LinkedOmics database, according to their ranking based on their Z-score through Spearman correlation analysis. GO BP (biological processes) analysis was obtained through gene ontology enrichment analysis performed on LinkedOmics, using the top 1000 KCNA3 positively correlated genes based on their *p*-value. Data were plotted as per fold enrichment, for the −log10 of the false-discovery rate (FDR) and the *p*-values, respectively.

Intrigued by the dual role played by *KCNA3* in tumors, which is associated with positive prognosis in SKCM (Figure 2F) and LUAD (Figure 2I) but not in LUSC (Figure 2J), we sought to identify *KCNA3* differentially expressed genes (DEGs) to clarify the functions with which it is involved (Figure 4A,C,E). To this end, genes positively correlated with *KCNA3* in SKCM, LUAD, and LUSC were identified from their respective TCGA datasets by classifying Z-scores and mapping them using the LinkedOmics database. Interestingly, several of the DEGs were common to at least two of the TCGA-tumor datasets taken into consideration. Furthermore, most of them were linked to immune activation mechanisms that take an active part in the fight against carcinogenesis. For example, *IKZF1* [60], *SLAMF1* [61,62], *ITGAL* [63,64], *C16orf54* [65], and *CYTIP* [66] are among the top 50 genes that are positively related to *KCNA3* and are present in all three types of tumors (Figure S2). These genes have all been reported to play a role in the regulation and/or recruitment of immune cells in tumors.

By classifying the top 1000 positively *KCNA3* co-expressed genes and listing them by gene ontology (GO) categories, we confirmed that *KCNA3*-positively related genes were involved in immune processes (Figure 4B,D,F). Indeed, some categories of biological processes (BPs) such as “*T cell activation*”, “*B cell activation*”, and “*Adaptive immune response*” were consistently found in all three datasets, suggesting a correlation between *KCNA3* expression and immune system regulation in cancer. Additionally, this role seems to be peculiar to *KCNA3* since *KCNA5*-related DEGs categories are not involved in these kinds of processes, as reported in Figure S3A,B.

### 3.6. KCNA3 Expression Correlates with Increased Immune Infiltration

**Figure 5:**
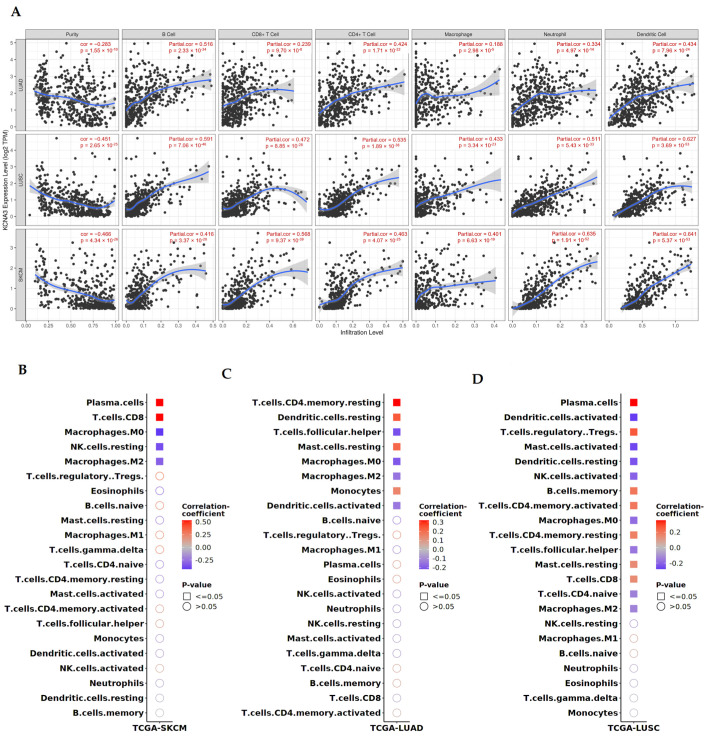
KCNA3 correlation with infiltrating immune cells and prognostic value in SKCM, LUAD, and LUSC. (**A**) Correlation between KCNA3 level of expression and immune infiltration cell types was obtained in the overall LUAD, LUSC, and SKCM TCGA dataset using the TIMER database. Significance and correlation values are shown. (**B**–**D**) Correlation between KCNA3 level of expression and immune infiltration in SKCM (**B**), LUAD (**C**), and LUSC (**D**), retrieved from the respective TCGA datasets employing the Survival Genie database tool. Significance and correlation coefficient are shown in the graphs.

To evaluate the possible involvement of *KCNA3* signaling in the regulation of the immune system, we decided to analyze the degree of immune cell infiltration in SKCM, LUAD, and LUSC tumors as a function of the ion channel expression (Figure 5A). In particular, we first employed the TIMER dataset to correlate *KCNA3* expression with the infiltration rate of different cell types when collected inside the TCGA cohorts, including B cells, CD8+ T cells, CD4+ T cells, macrophages, neutrophils, and dendritic cells. The analysis showed that the degree of infiltration for the immune subtypes always positively and significantly correlated with the level of *KCNA3* (Figure 5A). Tumor “purity” refers to a key element affecting the genomic analysis of immune infiltrates: noticeably, all the screened conditions showed negative correlations with such parameters.

Nevertheless, as observed in Figure 2, the prognostic role of *KCNA3* varied between the three examined tumors and was associated with a better prognosis in SKCM and LUAD only but not in LUSC. Considering the correlation with immune infiltrates, we hypothesized that *KCNA3* could exert a distinct immune recruitment function in the different tissues where it was expressed, thus representing an advantage or not for the tumor progression. To verify this hypothesis, we took advantage of the SurvivalGenie tool to analyse the positive or negative correlations between the immune cell subtypes and their active/resting forms and the expression of *KCNA3* (Figure 5B). Interestingly, in SKCM, a high level of *KCNA3* mRNA positively correlated with the elevated infiltration of CD8+ T cells, the immune cell type most associated with a greater immune response against the tumor and considered a positive biomarker of prognosis in melanoma (Figure 5B) [67]. Conversely, increased *KCNA3* transcript levels inversely correlate with M2 macrophage infiltration: a known marker of immune suppression (Figure 5B). Additionally, in LUAD, the expression of *KCNA3* seemed to be positively associated with the infiltration of immune subtypes that promote the anti-tumor response (Monocytes, CD4+ T cells, Mast cells, Dendritic cells) and are inversely related to M2 macrophages (Figure 5C).

Interestingly, in LUSC, where *KCNA3* correlates with a poor prognosis, the immune cell infiltration profile differs from the previous two cases (Figure 5D). For instance, high levels of *KCNA3* correlate with an increase in the infiltration of Tregs (Regulatory T cells): one of the main cell types linked to immune suppression. Moreover, elevated *KCNA3* levels were inversely correlated with the presence of dendritic cells (both active and resting), natural killers (NKs), CD4+ T cells, and mast cells (Figure 5D), which were all able to favor recognition, antigen presentation, and killing of tumor cells. These results suggest that the different composition of the *KCNA3*-related immune microenvironment in LUSC may favor mechanisms of immune evasion that could partially account for the different prognostic values of the gene in this tumor type.

## 4. Discussion

In recent years, ion channels have been studied intensively in the context of cancer since several pieces of evidence have appointed them as oncological targets [68,69]. In this paper, for the first time, we aimed to recapitulate all the available information collected in the publicly available databases concerning voltage-gated potassium channels and different types of cancers. In detail, our meta-analysis highlighted a possible correlation between KCNA potassium channels’ expression and cancer patients’ survival. We initially investigated KCNA gene alterations in several tumor types, demonstrating that SKCM, UCEC, STAD, LUSC, and LUAD are the five cancers with the highest KCNA mutation frequency. We further revealed that KCNA2, KCNA3, and KCNA5 as the most expressed KCNA family genes in these tumors. In addition, we were able to show a prognostic role for *KCNA3* expression in SKCM, LUAD, and LUSC and for *KCNA5* in STAD. Conversely, no correlation between *KCNA2* expression and patients’ survival has been observed in the analyzed cancer types. Therefore, we focused our observations on identifying a possible relation between KCNA3 and KCNA5 expression and patients’ clinical features and tumor risk factors, such as age, sex, obesity, and smoking. Some previous evidence suggested that KCNA3 plays a regulatory role in the processes of regulating body weight, fat absorption, and insulin sensitivity [70,71,72,73]. Although the reported data were controversial, it would be worth investigating these relationships in the context of melanoma, considering that KCNA3 associates with a better prognosis in this tumor type (Figure 2F). Recent evidence demonstrates that tobacco consumption strongly affects gene expression in smokers compared to non-smokers. Interestingly, among the genes whose expression was found to be altered, there was KCNA3 [74]. For this reason, a better assessment of the KCNA3 role in lung cancer should be carried out to investigate the difference in the positive prognostic role played by this ion channel in LUAD (Figure 2I) but not in LUSC (Figure 2J).

Nowadays, it is worldwide accepted that cancer development is supported by the tumor microenvironment (TME) [75]. In particular, TME cells (e.g., immune cells, CAFs, mesenchymal cells) interact with primary cancer cells and promote their ability to become invasive. Our data clearly show that a possible explanation of the differential prognostic role of KCNA3 could be related to its capability to contribute to the recruitment of immune cells in the TME. Indeed, we observed that in the tumor where KCNA3 exerts a positive role in increasing the survival rate when it is highly expressed (SKCM and LUAD), *KCNA3* mRNA expression is related to an elevated infiltration of anti-tumor immune cell sub-populations (e.g., CD8+ and CD4+ T cells, monocytes, dendritic cells), while the presence in the tumor microenvironment of M2 pro-tumorigenic macrophages was reduced. Conversely, in LUSC, where high KCNA3 expression is associated with poor prognosis, channel expression is correlated to higher pro-immune suppressive Tregs cell infiltration and a reduced presence of the anti-tumor immune cell sub-populations.

On the capability of potassium ions to modulate immune cell infiltration, Eil and colleagues have recently demonstrated that the increased extracellular potassium concentration due to the release from necrotic cancer cells plays a role in suppressing T cell functions within the tumor [76]. Do other immune cells have a similar checkpoint that modulates their function? Might cancer cells upregulate their levels of K^+^ channels to extrude potassium and survive in the potassium-rich extracellular fluid? These are important questions that need to be answered. On this line, according to our data, it would be important to understand how Kv1.3 could be modulated both in its expression and in its function. A possible signaling pathway that can act both on KCNA expression as well as regulating their activity could be linked to a burst in ROS [77]. The incomplete transfer of electrons to a specific target, along with the malfunction of antioxidant systems, could result in ROS production, which could lead to oxidative damage: a common phenomenon in most immune disorders. ROS are highly reactive molecules that can interact with various cellular constituents, such as DNA, lipids, and proteins, and therefore trigger cellular damage. In addition, the activation of mild oxidative stress may function as a second messenger in a signaling cascade promoted by alterations in the ion channel activity in response to hormones and neurotransmitters. Indeed, low oxidative stress is important for correct cell function [78]. Finally, it has also been demonstrated that openings or closures of mitochondria-located ion channels frequently lead to the modification of organelle functions that promote ROS release, for example, as observed with the inhibition of mitochondria-located Kv1.3 [35,79]. As for the immune system, hypoxia can impair the voltage-gated potassium channel (also Kv1.3) activity and expression, perhaps explaining the complications in proliferating of normally highly Kv1.3 expressing T-cells in hypoxic TME [80,81]. Similar to T-cells, the inhibition of Kv1.3 in macrophages leads to membrane depolarization and, in turn, the reduction in chemotactic migration [82] alongside the inhibition of stimulated proliferation and of the inducible nitric oxide synthase expression [83]. In addition, ROS are important mediators of pro-inflammatory signaling pathways, which modulate the expression of important transcription factors such as NF-kB and AP-1, that can support the up-regulation of pro-inflammatory chemokines/cytokines and adhesion molecules, which are also able to activate endothelial cells. In turn, endothelial cells can attract monocytes, which can then differentiate into macrophages [84]. On the other hand, ROS are also produced by immune cells to kill pathogens, but during long-lasting inflammation, this ROS-induced oxidative stress can damage endothelial cells [85]. In a scenario where the modulation of ROS could be beneficial to regulate immune responses to fighting cancer cells, a possible method of regulating ROS levels and antioxidant response is physical exercise, which can promote the release of stress hormones such as catecholamines and cortisol, resulting in the production of various cytokines, leading to either immune activation or immune suppression [85].

## 5. Conclusions

In conclusion, for the first time, we analyzed in detail the possible correlation of KCNA gene expression with patients’ survival in several tumors. Moreover, we demonstrated that a possible correlation in the case of Kv1.3 and Kv1.5 expression and some cancers’ prognosis exists. Finally, we observed a possible link between the prognostic role of Kv1.3 expression and its ability to recruit immune cells. In general, it is important to be aware of the limitation that by analyzing data obtained by datasets, we cannot discriminate between the *KCNA3* expression either in tumors or in the immune cells. Therefore, further work is necessary to dissect and clarify whether the observed KCNA3 expression affects prognosis by relating to cancer cell behavior or to the increased presence of Kv1.3 expressing immune infiltrates. In addition, the elucidation of the possible role of the redox state in tuning on one side ion channels expression and activity and on the other side immune cells proliferation and activation could point to an interesting molecular pathway that can be studied in cancer biology.

## Figures and Tables

**Table 1:** Co-expression analysis was carried out by two pearson correlation analysis between the *KCNA3* level of expression and the presented *KCNA3* interactor using cBioPortal SKCM (363 samples), LUSC (469 samples), and LUAD (507 samples) TCGA PanCancer Atlas datasets. Significant Spearman’s correlation values are reported in bold.

SKCM		
KCNA3 Interactor Gene Name	Spearman’s Correlation	*p*-Value
*DLG1*	0.128	0.0144
*DLG4*	0.0266	0.614
*ITGB1*	−0.0543	0.302
*CAV1*	−0.0111	0.833
*SEC24A*	0.249	1.507 × 10^−6^
*KCNE4*	0.367	5.5 × 10^−13^
*CTTN*	−0.300	5.62 × 10^−9^
**LUAD**		
**KCNA3 Interactor Gene Name**	**Spearman’s Correlation**	***p*-Value**
*DLG1*	−0.00292	0.948
*DLG4*	0.300	6.475 × 10^−12^
*ITGB1*	−0.211	1.7445 × 10^−6^
*CAV1*	−0.0205	0.646
*SEC24A*	0.181	4.4465 × 10^−5^
*KCNE4*	−0.0156	0.727
*CTTN*	−0.237	7.265 × 10^−8^
**LUSC**		
**KCNA3 Interactor Gene Name**	**Spearman’s Correlation**	***p*-Value**
*DLG1*	−0.18	9.7355 × 10^−5^
*DLG4*	0.259	1.485 × 10^−8^
*ITGB1*	0.165	3.4585 × 10^−4^
*CAV1*	0.0965	0.0372
*SEC24A*	0.469	8.015 × 10^−27^
*KCNE4*	0.422	1.475 × 10^−21^
*CTTN*	−0.0678	0.114

## Data Availability

Data is contained within the article or Supplementary Materials.

## Supplementary Materials

The following supporting information can be downloaded at: https://www.mdpi.com/article/10.3390/antiox12030573/s1, Figure S1. Analysis of the KCNAs transcriptional level. Figure S2. Correlation between KCNA3 level of expression with genes involved in cancer immune response in SKCM, LUAD, and LUSC. Figure S3. KCNA5 correlated differentially expressed genes and related pathways.
