# Supplementary material for: A Meta-Analysis Study to Infer Voltage-Gated K+ Channels Prognostic Value in Different Cancer Types
Source: Antioxidants (Basel). 2023 Feb 24;12(3):573. doi: 10.3390/antiox12030573 (PMC10045123; doi:10.3390/antiox12030573)
Supplement: Supplementary file 1 [file antioxidants-12-00573-s001.zip › antioxidants-2203735-supplementary.pdf]

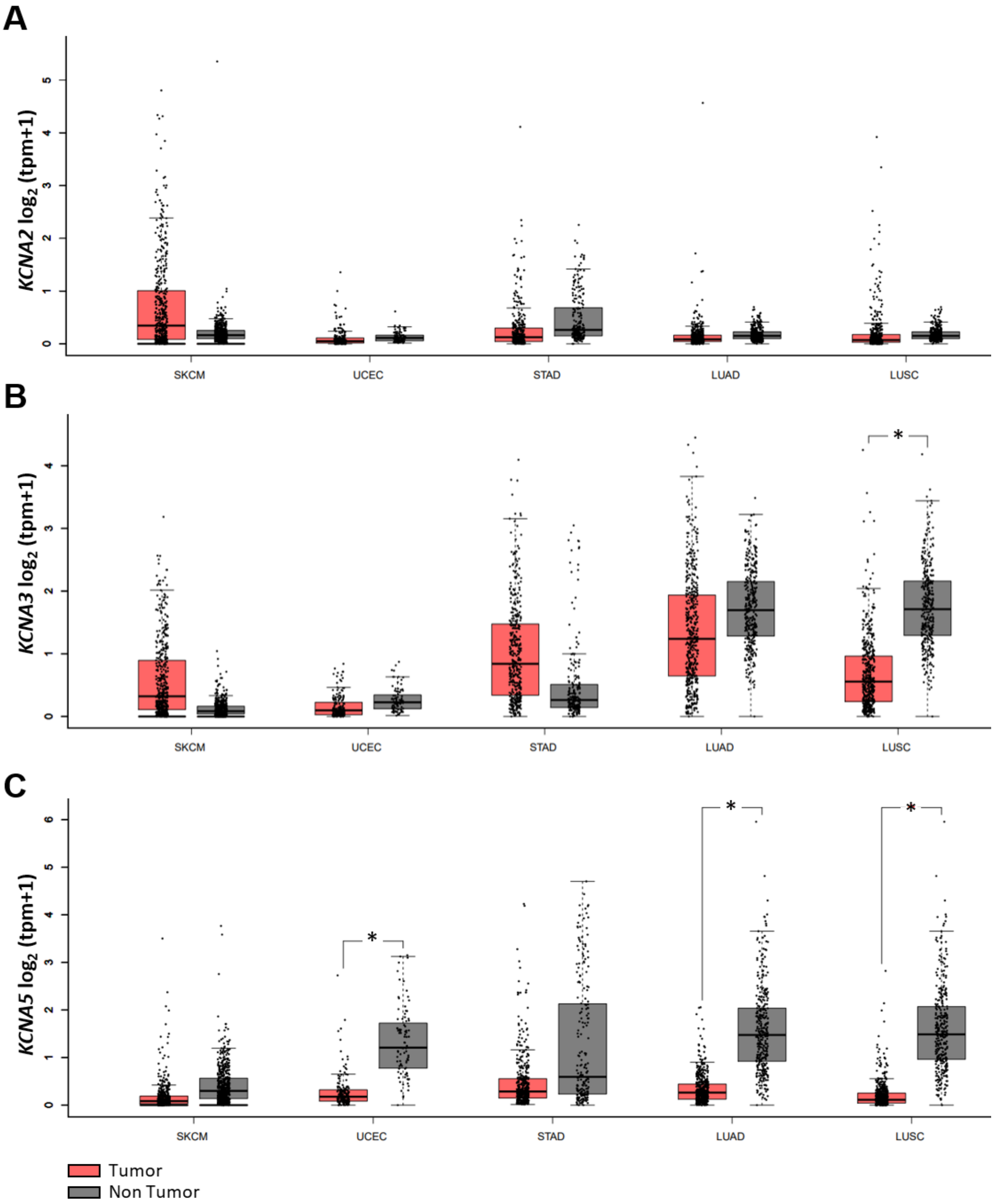

Figure S1. Analysis of the KCNAs transcriptional level. The mRNA expression of KCNA2 (A), KCNA3 (B) and KCNA5 (C) was assessed comparing tumor and normal tissues from TCGA and GTEz datasets using GEPIA database. Data were normalized as transcripts per kilobase million (TPM) values. TPM values were converted to log2-normalized transcripts per million [log2(TPM + 1)]. Data were shown as the mean ± standard deviation. Statistical analyses were performed using t-test. Error bars represented SD. * indicated p-value < 0.05.

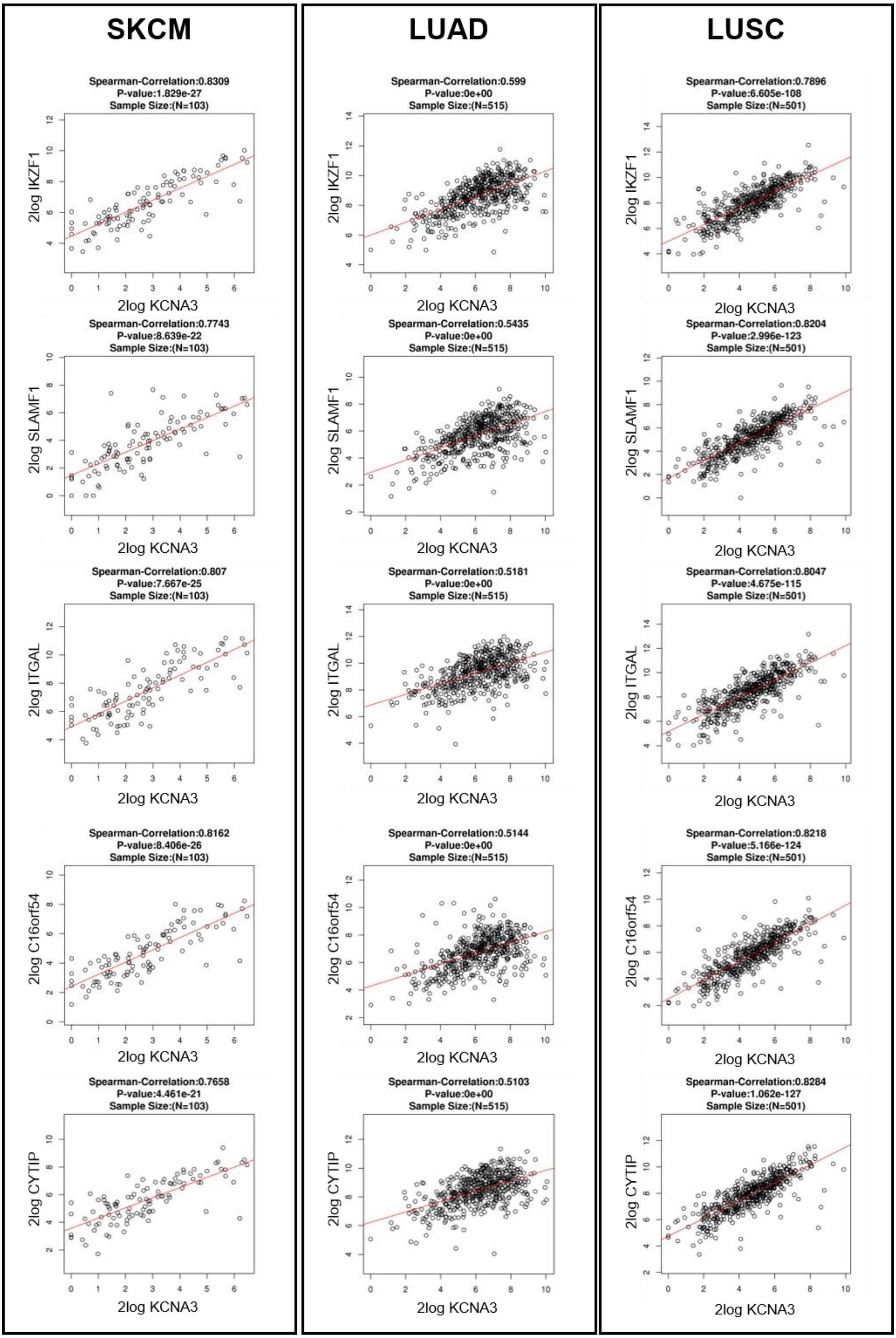

Figure S2. Correlation between KCNA3 level of expression with genes involved in cancer immune response in SKCM, LUAD, and LUSC. Two Pearson correlation analysis were obtained from LinkedOmics (* $p < 0.05$; ** $p < 0.01$; *** $p < 0.00$).

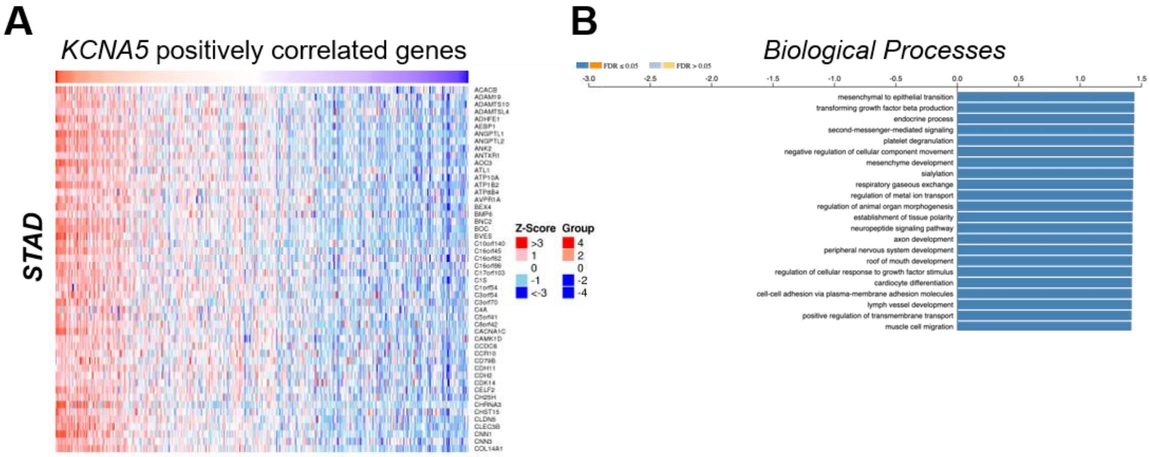

Figure S3. KCNA5 correlated differentially expressed genes and related pathways. (**A**) The top 50 positively KCNA5 coexpressed genes were mapped using the TCGA STAD dataset in the LinkedOmics database, according to their ranking based on their Z-score through Spearmann Correlation analysis. (**B**) GO BP (Biological Processes) analysis were obtained through Gene Ontology Enrichment Analysis performed on LinkedOmics, using the top 1000 KCNA5 positively correlated genes based on their p-value. Data were plotted as per fold enrichment, for the -log10 of the false-discovery rate (FDR) and the p-values respectively.
